# Retrospective reconstruction of four-dimensional magnetic resonance from interleaved cine imaging – A comparative study with four-dimensional computed tomography in the lung

**DOI:** 10.1016/j.phro.2023.100529

**Published:** 2023-12-27

**Authors:** Giulia Peteani, Chiara Paganelli, Anna Chiara Giovannelli, Barbara Bachtiary, Sairos Safai, Susanne Rogers, Orso Pusterla, Oliver Riesterer, Damien Charles Weber, Antony John Lomax, Guido Baroni, Giovanni Fattori

**Affiliations:** a Department of Electronics, Information and Bioengineering, Politecnico di Milano, Milan, Italy; b Center for Proton Therapy, Paul Scherrer Institut, Villigen, Switzerland; c Department of Physics, ETH Zürich, Zürich, Switzerland; d Department of Radiation Oncology, Kantonsspital Aarau, 5001 Aarau, Switzerland; e Department of Radiology, Division of Radiological Physics, University Hospital Basel, University of Basel, Basel, Switzerland; f Department of Radiation Oncology, University Hospital of Zürich, 8091 Zürich, Switzerland; g Department of Radiation Oncology, Inselspital, Bern University Hospital, University of Bern, Switzerland; h Bioengineering Unit, National Center of Oncological Hadrontherapy (CNAO), Pavia, Italy

**Keywords:** 4DMR, Lung cancer, Organ motion, Radiotherapy

## Abstract

**Background and purpose:**

Imaging of respiration-induced anatomical changes is essential to ensure high accuracy in radiotherapy of lung cancer. We expanded here on methods for retrospective reconstruction of time-resolved volumetric magnetic resonance (4DMR) of the thoracic region and benchmarked the results against 4D computed tomography (4DCT).

**Materials and method:**

MR data of six lung cancer patients were collected by interleaving cine-navigator images with 2D data frame images, acquired across the thorax. The data frame images have been stacked in volumes based on a similarity metric that considers the anatomical deformation of lungs, while addressing ambiguities in respiratory phase detection and interpolation of missing data. The resulting images were validated against cine-navigator images and compared to paired 4DCTs in terms of amplitude and period of motion, assessing differences in internal target volume (ITV) margin definition.

**Results:**

4DMR-based motion amplitude was on average within 1.8 mm of that measured in the corresponding 2D cine-navigator images. In our dataset, the 4DCT motion and the 4DMR median amplitude were always within 3.8 mm. The median period was generally close to CT references, although deviations up to 24 % have been observed. These changes were reflected in the ITV, which was generally larger for MRI than for 4DCT (up to 39.7 %).

**Conclusions:**

The proposed algorithm for retrospective reconstruction of time-resolved volumetric MR provided quality anatomical images with high temporal resolution for motion modelling and treatment planning. The potential for imaging organ motion variability makes 4DMR a valuable complement to standard 4DCT imaging.

## Highlights

• Stacking of 2D cine can generate 3D MR images at high temporal resolution (4DMR).
• Motion amplitude measured in lung with 4DMR and cine differed by less than 1.1 mm.
• 4DCT amplitude was generally within 3.8 mm of the MR-based figures.
• Variations up to 24% of the 4DCT breathing period were observed in 4DMR datasets.
• 4DMR offers unique data for refining treatment volumes with probabilistic approaches.

## 1 Introduction

Breathing is the main cause of organ motion in the thoraco-abdominal area, which has an impact on planning and delivery of radiotherapy treatments [1]. In case of moving targets due to patient’s breathing, time-resolved (4D) imaging is fundamental to model the organ motion and put in place the necessary countermeasures to ensure treatment precision [2], [3].

The standard imaging modality for organ motion management in radiotherapy is 4D Computed Tomography (4DCT) [4]. As 4DCT provides only an average cycle, it is questionable whether this is representative of patient breathing throughout the fractions or informative enough for delineating target margins for radiotherapy treatments [5]. These concerns are relevant in proton therapy, a treatment modality whose precision is called into question in the presence of anatomical changes [6] or intra-fractional organ motion [7], such as the respiration.

In recent years, therefore, there has been a growing interest in the use of Magnetic Resonance Imaging (MRI) [8], [9]. The absence of imaging dose allows capturing cycle-to-cycle breathing variations during extended sessions [10], [11], for supporting clinical decisions and treatment planning [12]. Several studies employing 2D cine-MR have shown large differences in tumour motion and cycle-to-cycle variations with respect to 4DCT measurements [13], [14]. However, cine-MR alone cannot fully cover the needs of proton therapy, which requires accurate 3D information about tumour position and any upstream tissues in the treatment fields [9].

With the current technology, well-contrasted MR volumes can only be acquired at low sampling rate [15], and therefore rapidly acquired 2D slices need to be sorted and stacked retrospectively. Several approaches have been proposed for 4DMRI reconstruction [8], [16], typically involving the simultaneous acquisition of a motion surrogate during imaging and its later use for stacking images into a limited number of breathing phases. More sophisticated methods implement time-resolved 4DMRI by combining respiratory correlated 4DMRI workflow with dynamic 2D cine [17], [18], with a demonstrated use in the imaging of liver and the possibility to obtain continuous 3D volumes over several respiratory cycles. Alternative approaches to directly derive time-resolved 3D volumes were investigated in the literature, such as dedicated k-sampling strategies with compressed sensing [19], super resolution techniques [20] or motion modelling [21]. However, all these strategies require ad-hoc acquisition schemes in the MR scanner, which are not always available in clinical scanners, and dedicated prediction models to estimate 3D motion.

In this study, we describe a novel approach for reconstructing 4DMRI of the lung region, with the aim of modelling anatomical changes occurring over several minutes of acquisition as continuous respiratory-correlated 4DMRI. Specifically, we expanded on the work of von Siebenthal et al [17], with which we share the same MR imaging sequence. Our approach applies on lung imaging, which involves stacking images whose position may be distant from the navigator slice used to define the respiratory surrogate, and the non-symmetrical motion of the two lungs is a confounding element when stacking images of the contralateral lung relative to the navigator. Therefore, we reworked the stacking approach to handle ambiguities in the selection of data slices constituting volumes and the lack of candidates within a similarity metric threshold. Furthermore, with regard to lung imaging, the template matching process in the original publication has been replaced with the analysis of the deformation vector field across the entire lung region for better modelling of organ deformation. 4DMRI of six lung cancer patients were compared with paired 4DCT images in terms of amplitude and period of motion, to benchmark the 4DCT-based clinical standard in terms of motion analysis and definition of treatment volumes.

## 2 Materials and methods

### 2.1 Patient dataset and imaging protocol

MR of six lung cancer patients under treatment at the radiotherapy department of Kantonsspital Aarau (KSA) in Switzerland were acquired at the Center for Proton Therapy of Paul Scherrer Institute (Switzerland), with planning 4DCT images collected at KSA. All patients have signed informed consent and data were acquired within the frame of the EKNZ 2014-194 prospective imaging study, approved by the responsible ethics committee. Gross tumour volumes (GTV) and clinical target volumes (CTV) were defined by an experienced radio-oncologist following ESTRO guidelines [22] on the mid-position (MP) image [23] of the 4DCT dataset of each patient.

4DCT images were acquired on a SOMATOM scanner (Siemens Healthineers, Erlangen, Germany) operating at 120 kVp in helical mode with a Varian Real-time Position Management system (RPM) with an in-plane pixel spacing of 0.97 × 0.97 mm and 1 mm slice thickness. The MR data were acquired with a 1.5 T MAGNETOM Aera MR system (Siemens Healthineers, Erlangen, Germany) using a custom-made balanced Steady-State Free Precession (bSSFP) sequence with cartesian k-space sampling [24], [25]. The 4DMR was based on interleaved acquisition of cine-MR data at a fixed anatomical position, called navigators, with moving data frames acquired across the thorax [17], covering the required field of view (FOV) repeatedly. In this process the navigator image plane doesn’t overlap with any data frames and thus forms an independent dataset. Each acquisition lasted about 5–6 min using the following parameter set for both navigators and data frames: sagittal orientation; TE = 0.81 ms; TR = 1.75 ms; flip angle = 55°; image slice thickness is of 4 to 6 mm and in-plane pixel spacing of 1.01 × 1.01 mm^2^.

### 2.2 Retrospective sorting of 2D images and 4D volume reconstruction

**Fig. 1:**
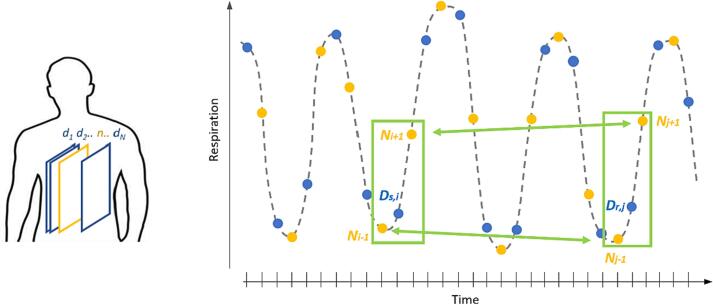
Acquisition of data frames (blue) and navigators (yellow) over time (3.5 ms between consecutive navigators/data frames, and 1.75 ms between one navigator and the following data frame. The first image acquired defines time zero. A volume is reconstructed for each data frame acquisition timestamp.

Stacking 2D images into 3D volumes required to find data acquired under similar anatomical conditions to be associated in a volume. Since data frames forming the volume were acquired while sweeping across the imaging FOV and hence far apart in time, navigators were used to identify the breathing state, thus comparing images on the same anatomical plane. Particularly, correspondence of data frames was determined by comparing their neighbouring navigators in the interleaved sequence (Fig. 1). For that, after equalising the raw data intensity histogram, 2D deformable image registration (DIR) between all navigators and the first one acquired was calculated using L2L1 optical flow [26], with a weighting factor of 0.6 and a coarse-to-fine strategy, from a 4-fold down sampled image to its original resolution. The refinement within each resolution level was performed only once.

With reference to Fig. 1, consider a data frame D_s,i_ acquired at a slice position *s* and time *i*, its enclosing navigators N_i-1_ and N_i+1_ were compared with the corresponding navigators N_j-1_, N_j+1_ of any other data frame D_r,j_ acquired at the slice position *r* in time *j,* to identify the most similar ones. Weighted Mean Square Error (*wMSE*) for all voxels of the 2D deformable vector fields within a patient-specific region of interest (ROI) manually defined on the lung area [27], was used as similarity measure. With *n* being the number of voxels (*p)* belonging to such a ROI, wMSE was defined as follows:

(1) $$\mathrm{wMSE}=\frac{1}{n}\frac{\sum_{p=1}^{n}w_{p}{\left(y_{p}-\hat{y_{p}}\right)}^{2}}{\sum_{p=1}^{n}w_{p}}$$

as the weighted ($w_{p}$) sum of the squared differences between the displacement vectors yp,yp^ of corresponding voxels in the navigators’ ROI, with $y_{p}$ being the vector associated to the pixel *p* of navigator N_i-1_ and $\hat{y_{p}}$ the vector associated to the corresponding pixel in any other navigator.

*wMSE* was computed individually for the superior-inferior (SI) and anterior-posterior (AP) motion components, before adding them into one similarity figure. The contribution of each voxel *p* was weighted by considering its motion amplitude during the whole acquisition, individually for SI and AP, as described in Supplementary 1.1.

To identify data frames similar to D_s,i_, the similarity measure *c(i,j)* between pairs of embracing navigators was computed for each possible pair of data frames D_s,i_ and D_r,j_:

(2) $$c\left(i,j\right)=\left|\left[wMSE\left(i+1\right)-wMSE\left(j+1\right)\right]+\left[wMSE\left(i-1\right)-wMSE\left(j-1\right)\right]\right|$$

The normalized similarity was used to identify for each slice location *r* in the volume V_i_, the three candidate data frames with the lowest metric.

If not similar, data frames were discarded by setting an upper bound in the *c(i,j)* that was adapted to the specific breathing phase to be reconstructed. To automatise the threshold definition, the cumulative distribution of the percentage of slice locations in the volume for which at least one data frame candidate can be identified has been evaluated. This threshold has been defined such that more than one third of slice positions in the volume had at least one candidate data frame. In addition, an upper bound threshold cut was set empirically at 0.09, to ensure a minimum level of acceptable similarity.

**Fig. 2:**
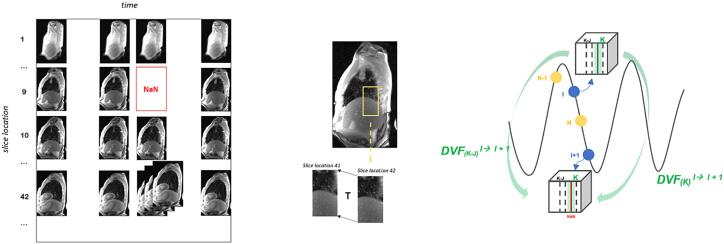
Condition of multiple matches, as for slice location 42, or absence of candidates to fill all slice locations in the volume, as for location 9. Frames selection and slice interpolation workflow are represented.

Once all data frames were processed, 4DMR volumes were reconstructed as stacks of slices (Fig. 2).

For each slice location in a volume, there could have been multiple data frame candidates within the similarity threshold. To select the best image, the candidate with minimum displacement with respect to the adjacent slice was selected (Supplementary 1.2).

On the other hand, due to the upper bound threshold cut, sometimes no data candidates for a given slice location were available. In this case, slice interpolation was performed to fill the vacancy with synthetic images, if at least 50 % of slices were available. Implementation details are reported in Supplementary 1.3.

### 2.3 Validation and comparison with the state of the art

The 4DMRI reconstruction has been validated by comparison with ground truth cine-MR navigator images by extracting in-plane motion at the image plane corresponding to the navigators from the 3D vector fields resulting from DIR [28] between the first and subsequent volumes (details reported in Supplementary 1.4). The reference deformation was computed from cine-MR navigators using the same settings as described in section 2.2. The difference between the two planar vector fields was evaluated to assess the reconstruction error in the lung, providing the median and interquartile range of the calculated difference for all ROI’s voxels.

Additionally, the proposed method has been compared with a reference stacking approach for the retrospective reconstruction of 4D MR images of an average breathing cycle using external surrogates [29]. The comparison and the related results are reported in Supplementary 1.5.

### 2.4 Breathing motion parameters

The reconstructed 4DMR datasets were used to test how representative 4DCT is for the key respiration parameters of motion amplitude, period and drift. For each patient, the motion of two points, on the tumour and diaphragm, has been tracked using 3D DIR computed as described in section 2.3. The distribution of respiratory peak prominences over time has been compared with the displacement of the same points between end-exhale and full-inhale on the 4DCT. Similarly, the time interval between subsequent end-exhales on the MR data has been compared with the average breathing period measured during the 4DCT scan of the patient using the RPM system. Finally, the baseline drift of the tumour and diaphragm in their main motion direction was measured on 4DMR data as the difference between its position in the first end-exhale phase and the subsequent ones.

### 2.5 Evaluation of ITV

In radiation therapy, the Internal Target Volume (ITV) is commonly based on treatment planning 4DCT imaging of an average breathing cycle and 4DMR has potential for including a larger range of variability in its definition. Therefore, using reconstructed 4DMRs, two ITV’s have been defined for each patient. First an ITV_4DCT_ as the union of the CTVs in all respiratory phases of the 4DCT dataset following DIR-based propagation. Second, the deformable motion computed from the 4DMRI was used to warp the mid-position image of each patient 4DCT, generating synthetic 4DCT(MR) datasets [30] to define the ITV_4DMR_, which includes the breathing variability observed during MR imaging. ITV_4DCT_ and ITV_4DMR_ have been compared using Dice coefficient, Hausdorff distance, centres of mass (COM) distances and their volume relative difference (ΔV%).

## 3 Results

### 3.1 4D volume reconstruction

Overall, it was possible to reconstruct MR volumes for more than 98 % of the navigator images’ time stamps, except for a few instants characterised by large respiratory singularities. On average, between 3.2 % and 12.4 % of the volumes consisted of synthetic images that made up for the lack of sufficiently redundant original data to stack. Exemplary 4DMR images of patient P3 available as supplementary material together with the paired 4DCT dataset.

### 3.2 Validation

**Fig. 3:**
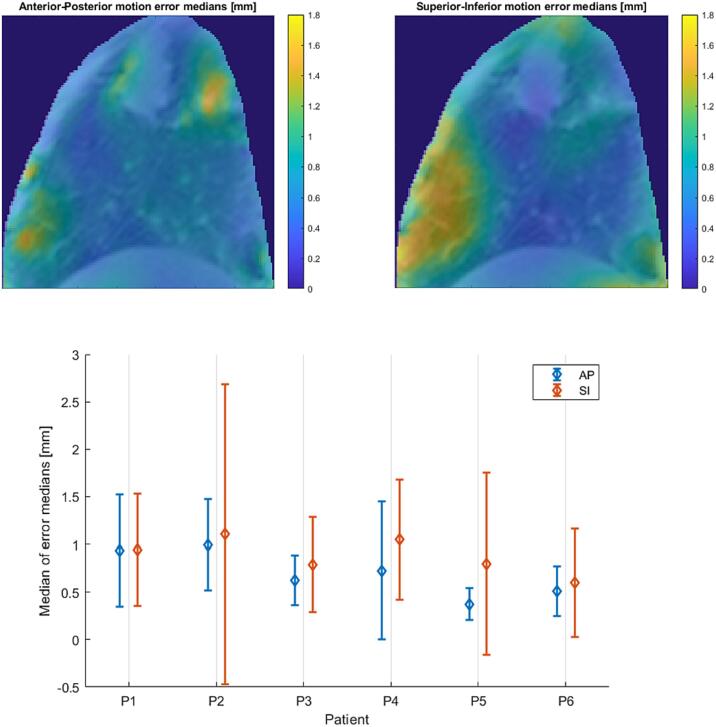
Error map (above) computed for AP and SI components for patient P3 and error bar plot (below) showing the median and interquartile range of the median errors in the lung ROI of each patient.

The proposed method has proven capable of reconstructing 4DMR volumes that can capture lung organ motion to an extent similar to that observed by 2D tracking in cine-MR navigator images. The median of the differences between the 4DMR and ground truth navigator imaging along the AP direction was between 0.4 and 0.9 mm, and slightly higher for SI, with a maximum around 1.1 mm as shown in Fig. 3. Furthermore, with an interquartile range within 1.5 mm the 4DMR volumes ensured consistent accuracy of reconstructed motion in the entire lung region, although errors were localised in feature-rich regions with larger movements (Fig. 3). The 95^th^ percentiles of the differences are reported in Supplementary 1.4.

### 3.3 Breathing amplitude and period

**Fig. 4:**
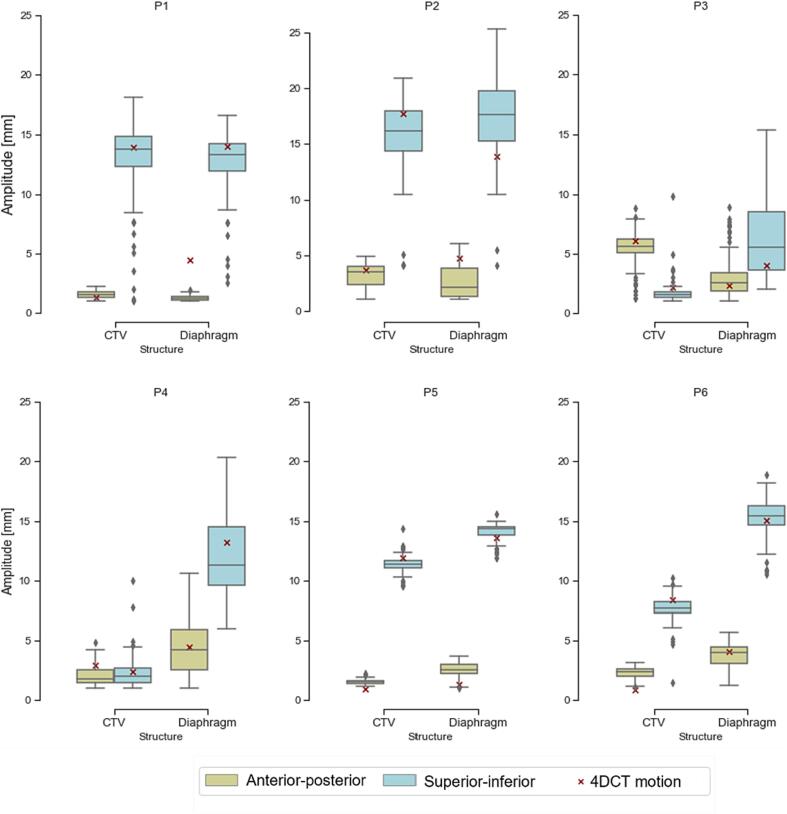
Comparison between motion amplitude distribution.

The distribution of breathing amplitudes is shown in Fig. 4 for the CTV and one point in the diaphragm region for all patients in our cohort. Going from apical (P3, P4) to the medial (P6, P2) and inferior (P5, P1) lesions, the motion turned from being primarily in AP to SI.

**Fig. 5:**
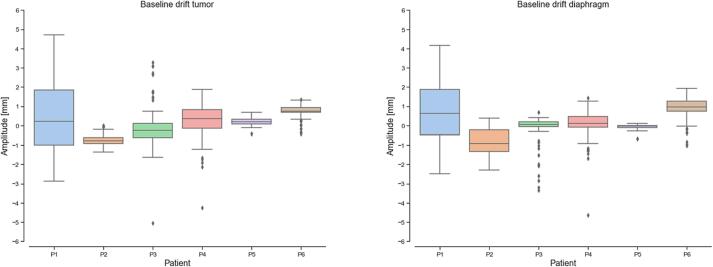
Distribution of end-exhale drift of tumour and diaphragm with respect to the first cycle reconstructed.

4DCT amplitude estimates were most often within a few millimetres (within 3.8 mm for CTV) of the median MR-based measure, except for patients with very large motions (>15 mm), where larger mismatches were observed between the two, especially in the diaphragm region. However, these combined with baseline drifts, which in our cohort had a mean ± standard deviation of 0.2 ± 1 and 0.2 ± 1 mm for diaphragm and tumour respectively in the main motion direction (Fig. 5). Breath-to-breath end-exhale position varied up to 5 mm. The breathing period computed during CT scans was comparable with that estimated from the 4DMRI in four patients out of six (Supplementary 1.6). In the other two cases the difference was as high as 24.1 %.

### 3.4 Evaluation of ITV differences

**Fig. 6:**
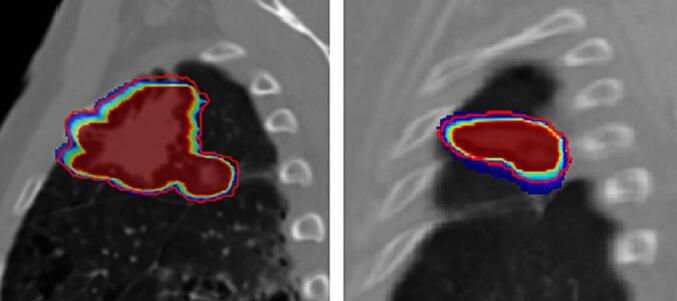
Example of probabilistic-ITV computed for patient P3 and P4 and their overlapping with the conventional 4DCT-ITV delineated by the red line.

Overall, ITV_4DCT_ and ITV_4DMR_ compared relatively well (Fig. 6). Dice coefficients between the two were high (0.9), and similarly the position of their COM was on average (std.dev.) within 2.1 mm (1.3 mm) distance. The magnitude of ΔV% was observed to be highly sensitive to motion amplitude variability (P2) and motion outliers (P4). The detailed list of metrics for all patients is reported in Supplementary Table S2.

## 4 Discussion

Several refinements in retrospective reconstruction of 4DMRI of the lung from interleaved cine-MR imaging have been introduced. This organ is characterised by a variety of motion between lobes, with a change in primary direction and breath-to-breath variability. These conditions lead to scarce raw data availability [10], under sampling the patient’s volume over time. The proposed algorithm includes the generation of synthetic images to complete partial volumes, frequent in irregular breathers, and the extraction of a robust image-based respiratory surrogate for the labelling of similar breathing anatomies throughout the acquisition. In this latter aspect, we expanded on commonly used methods that rely on template matching of a small ROI on the diaphragm [10], with the entire lung region, thus ensuring consistent reconstruction results across the whole volume. As a matter of fact, the quality of the reconstruction strongly depends on that of the surrogate information used for labelling corresponding breathing states in the time series and in such a complex patient site, the liver-lung interface is an overly simplistic representation of overall organ deformation. In addition, a distinct aspect concerned the use of voxel-specific weights and similarity thresholds dependent on the respiratory state to be reconstructed, ensuring reconstruction quality. Therefore, the incorporation of these refinements empowered the model to furnish essential insights into the tumour's motion and its variability. This aspect, notably absent in 4DCT-based workflows, as highlighted by the conducted comparison, can support enhanced precision in treatment planning.

The comparative analysis of motion characteristics from 4DMR and 4DCT confirmed that 4DCT might not always be exact, but in general it’s a reasonable approximation for both breathing amplitude and period. However, the respiratory variability can lead to substantially different cycles that challenge the use of 4DCT alone in the context of high-precision radiotherapy. In fact, the breath-to-breath drift in the end-exhale position, of the order of a few millimetres, may be of concern in some radiotherapy settings, as respiratory gating.

Imaging of motion variability is the real advantage of 4DMRI and hence its potential to extend the 4DCT motion description to support clinical decisions [8]. In the context of a clinical application, synthetic time-resolved CT images have been generated and used to investigate an alternative definition of the ITV for treatment of lung cancer. Although the differences in position and shape were relatively small between 4DMR and 4DCT-based ITVs, the discrepancies in relative volume size reflecting the extent of motion variability are of interest. In this context, 4DMR can provide unique data to refine the ITV with probabilistic approaches [31], [32], [33].

In conclusion, this study is a contribution to the field of 4D imaging for motion modelling and treatment planning of high-precision radiotherapy treatments of lung cancer. The proposed algorithm for retrospective reconstruction of MRI volumes proved capable to provide quality anatomical images with high temporal resolution. Although further analyses are required on a larger population, our results show how 4DMRI can be reconstructed and support standard CT imaging in a clinical workflow.

## CRediT authorship contribution statement

**Giulia Peteani:** Conceptualization, Methodology, Formal analysis, Investigation, Writing – original draft. **Chiara Paganelli:** Conceptualization, Methodology, Writing – review & editing, Supervision. **Anna Chiara Giovannelli:** Methodology, Investigation. **Barbara Bachtiary:** Resources, Data curation. **Sairos Safai:** Conceptualization, Funding acquisition. **Susanne Rogers:** Resources, Data curation. **Orso Pusterla:** Resources, Software. **Oliver Riesterer:** Resources, Data curation. **Damien Charles Weber:** Conceptualization, Resources, Funding acquisition. **Antony John Lomax:** Conceptualization, Supervision, Funding acquisition. **Guido Baroni:** Conceptualization, Supervision. **Giovanni Fattori:** Conceptualization, Methodology, Writing – review & editing, Supervision, Funding acquisition.

## Declaration of competing interest

The authors declare that they have no known competing financial interests or personal relationships that could have appeared to influence the work reported in this paper.
