# Supplementary figures and images for: Retrospective reconstruction of four-dimensional magnetic resonance from interleaved cine imaging – A comparative study with four-dimensional computed tomography in the lung
Source: Phys Imaging Radiat Oncol. 2023 Dec 27;29:100529. doi: 10.1016/j.phro.2023.100529 (PMC10792758; doi:10.1016/j.phro.2023.100529)

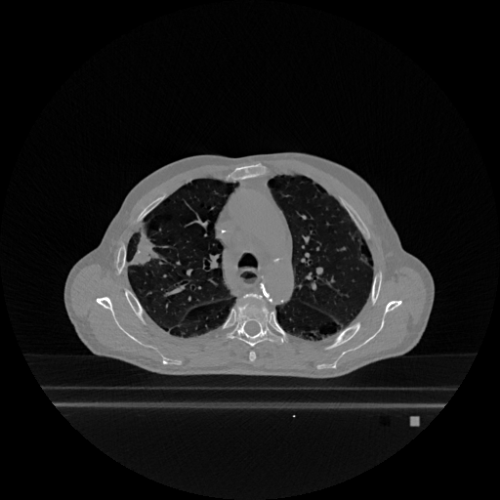

Supplement: Supplementary video 2 [file mmc2.gif]

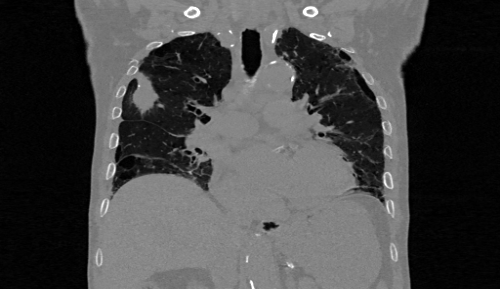

Supplement: Supplementary video 3 [file mmc3.gif]

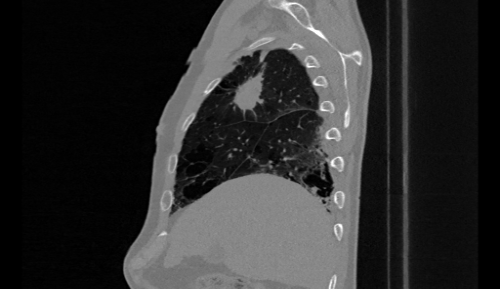

Supplement: Supplementary video 4 [file mmc4.gif]

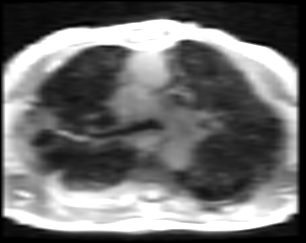

Supplement: Supplementary video 5 [file mmc5.gif]

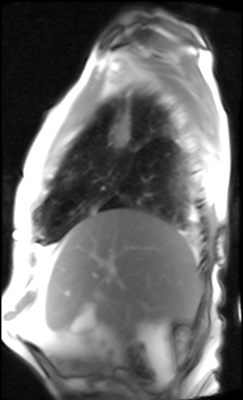

Supplement: Supplementary video 7 [file mmc7.gif]
