# Supplementary material for: Retrospective reconstruction of four-dimensional magnetic resonance from interleaved cine imaging – A comparative study with four-dimensional computed tomography in the lung
Source: Phys Imaging Radiat Oncol. 2023 Dec 27;29:100529. doi: 10.1016/j.phro.2023.100529 (PMC10792758; doi:10.1016/j.phro.2023.100529)
Supplement: Supplementary data 1 [file mmc1.docx]

SUPPLEMENTARY MATERIAL

**1.1 Weights computation**

The contribution of each voxel *p* to the *wMSE* (along SI and AP respectively) is weighted by considering its motion amplitude in the whole acquisition, individually for the two motion components, as follows:

$$w_{SI}=H*W_{SI} with W_{SI}=norm\left( \frac{\left[ {vf}_{SI} \right]_{90th}}{\left[ {vf}_{AP} \right]_{90th}} \right)$$

(Suppl E.1)

$$w_{AP}=H*W_{AP} with W_{AP}=norm\left( \frac{\left[ {vf}_{AP} \right]_{90th}}{\left[ {vf}_{SI} \right]_{90th}} \right)$$

(Suppl E.2)

$$H=\left\{ \begin{matrix} 0, W<Thr \\ 1, W\geq Thr \end{matrix} \right.$$

(Suppl E.3)

The individual component weight ($w_{SI}$or $w_{AP}$) is defined as the ratio of the 90^th^ percentiles of voxel’s deformation magnitude over time (Suppl E.1, Suppl E.2). This definition of weights is such to emphasise the larger motion component in each voxel, while making secondary motion even smaller. In a final step, the weights are normalised throughout the whole series of weights computed for the navigator and after the application of a lower boundary threshold ($Thr$ in Suppl E.3), which has been empirically set at 0.3.

**1.2 Frame selection**

The criteria to select the best candidate consists of minimising image differences among neighbouring data slices. A rectangular region of interest is manually identified centred on the diaphragm region, an operation that for simplicity is done on one of the corresponding navigator slices. Then, the three candidate data frames are evaluated within the same ROI boundaries to estimate the diaphragm displacement with respect to the adjacent slices for the same time frame. The rigid registration within the ROI in the slice at s position, ROI(s), and that in the slice at the position s-1, ROI(s-1), is computed using Matlab inbuilt function ‘imregtform’ that uses the regular step gradient descent optimizer and mean squares as metric. The superior-inferior component of the transform is considered to choose the best data frame among the candidates the image selected, as the one with the minimum translational component.

If multiple candidates are identified for the first slice position 0, the best data frame to stack is selected according to the minimum similarity metric *c(i,j).*

This selection method is designed to ensure, whenever possible, the continuity and smoothness of contours of largely moving organ boundaries. To limit the reconstruction computational time, the choice is limited to the three best matching data frames, hence it assumes that among such best candidates there is at least one with a small displacement with respect to the adjacent slices.

Lastly, it should be mentioned that the chosen method for selecting these slices might introduce a bias when the anatomy of the neighbouring slices is different, as at the borders of distinct anatomical regions, like where the spinal column meets the lung tissue. Nevertheless, it's worth noting that this study focuses specifically on lung imaging, so this potential bias doesn't concern the area of interest.

**1.3 Frames interpolation**

Considering a volume D_i+1_ corresponding to the data frame D_s,i+1_ acquired at time i+1 and a missing slice at the K position in that volume (Figure S.1), the interpolation procedure begins by searching in the neighbourhood of K the closest slice position K-J at which a slice is available in the I+1 volume, D_k-j, i+1_, and in the previous one I, D_k-j, i_ . This search is limited to the four previous and following slice positions of K. Afterwards, DIR is computed between D_k-j,i_ , set as the moving image, and fixed D_k-j,i+1_. The resulting deformation vector fields (DVF^K-J^ I → I+1) describe the displacement of each voxel in the moving D_k-j,i_ to warp into the corresponding voxel in the fixed D_k-j,i+1_. DIR is calculated based on the optical flow between the two images, D_k-j,i_ and D_k-j,i+1_, using the same tools and settings presented in 1.1. Under the assumption the geometry transform between consecutive time instants is similar for contiguous slices, such DVF^K-J^ I → I+1 is used to warp the slice at the position K in the volume I and obtain the slice at the position K in the volume I+1, which was missing.


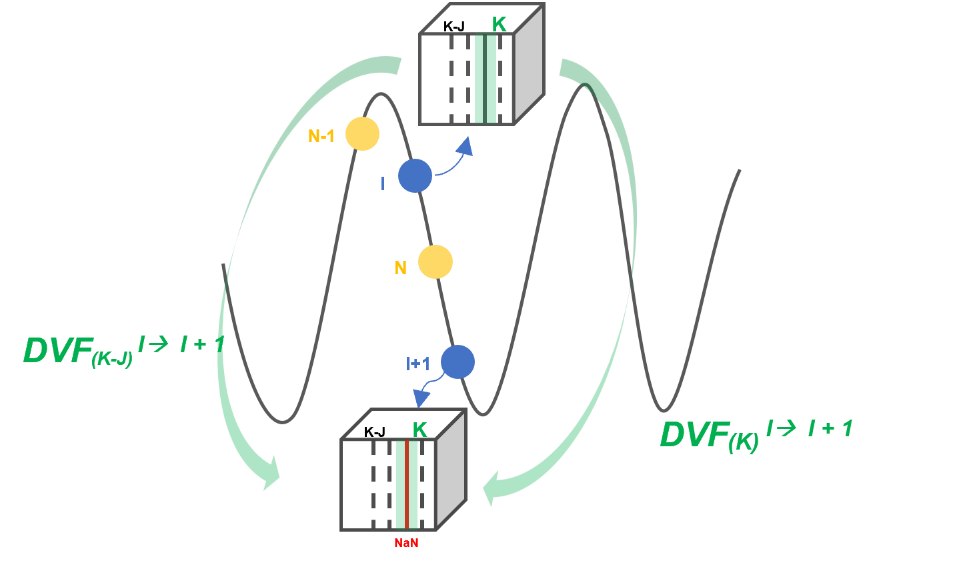


Figure S.1: Slice interpolation workflow

**1.4 Validation**

Multi-resolution B-spline registration was applied after an initial rigid alignment (Plastimatch [28]). Three registration stages were optimized with the limited memory Broyden–Fletcher–Goldfarb–Shanno algorithm (LM-BFGS) optimizer, starting from a coarse resolution and B-spline grid spacing, to a finer one at original voxel size with a regularization parameter of 0.005.

It should be noted that the reliability of the reconstruction is proven by comparing the 4DMRI volumes with the ground truth of the cine-MR navigator images using two different DIR algorithms, optical flow based on 2D images and b-spline based on 3D volumes. Although all DIR methods require careful quality assurance in the perspective of a clinical application, the use of different algorithms is a strength of this comparison, not being biased by the uncertainty of a single approach.

The 95^th^ percentile of the differences between the 4DMR and ground truth cine imaging along AP direction, as worst-case scenario in our dataset, is between 0.9 and 1.9mm, and slightly higher for SI, that present a maximum as high as 3.6mm.

|  | **P1** | **P2** | **P3** | **P4** | **P5** | **P6** |
| --- | --- | --- | --- | --- | --- | --- |

| **AP PRCTILE [mm]** | 1.9 | 1.8 | 1.0 | 2.0 | 0.9 | 1.4 |
| --- | --- | --- | --- | --- | --- | --- |
| **SI PRCTILE [mm]** | 1.7 | 3.6 | 1.8 | 2.2 | 2.7 | 1.8 |

Table S1: 95^th^ percentile of the median errors, relative to the anterior-posterior (AP) and superior-inferior (SI) components, computed in the lung ROI of each patient.

## **1.5 Comparison with the state of the art**

The proposed method has been compared with a reference stacking approach for the retrospective reconstruction of 4D MR images of an average breathing cycle using external surrogates [29]. The comparison is based on the digital MRI phantom data p0302 from the original publication [29], featuring clinically relevant motion amplitudes and variability (the diaphragm’s range of motion is 19.9mm, and the tumour’s one is 3.7mm and 7.3mm along AP and SI directions respectively; the mean period 3.7s). The 4DMR datasets resulting from both reconstruction algorithms have been processed to assess the tumour motion using 3D DIR, following the same approach described in section 2.3. In particular, focusing mainly on motion amplitude, each breathing cycle output from the hereto proposed method has been normalised to its period to evaluate the difference to the reference average motion described by the original method.

The average breathing cycle obtained with the reference stacking algorithm [29] and cycles reconstructed with the method introduced in this paper are shown in figure S.2. The difference between the median tumour position obtained from all reconstructed breaths and the reference curve varies with the breathing, going from a minimum of 0.12mm at the end-exhale up to 0.34mm at the full inhale phase. Moreover, larger variability is observed in inhale positions, manifesting as an interquartile range of motion amplitude reaching up to 1.11 mm.


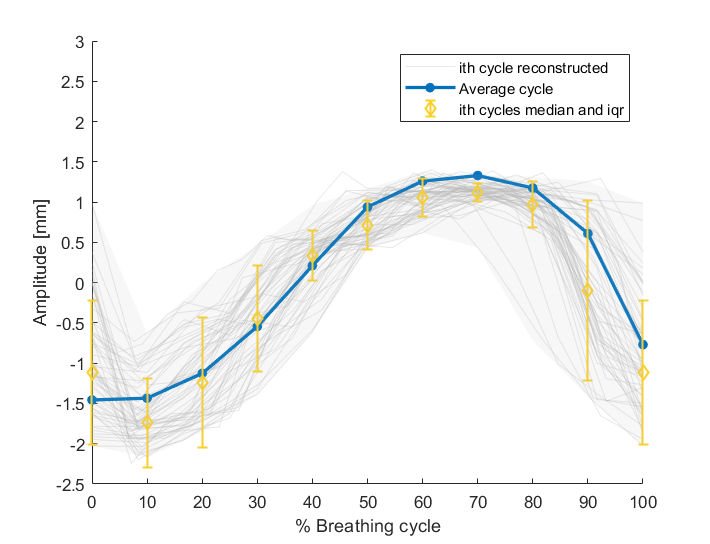


*Figure S.2: Comparison between the average breathing cycle reconstructed with the reference algorithm and the cycles reconstructed with the proposed one. Median and interquartile range of the tumor position among the cycles are reported for each breathing phase.*

**1.6 Breathing period**


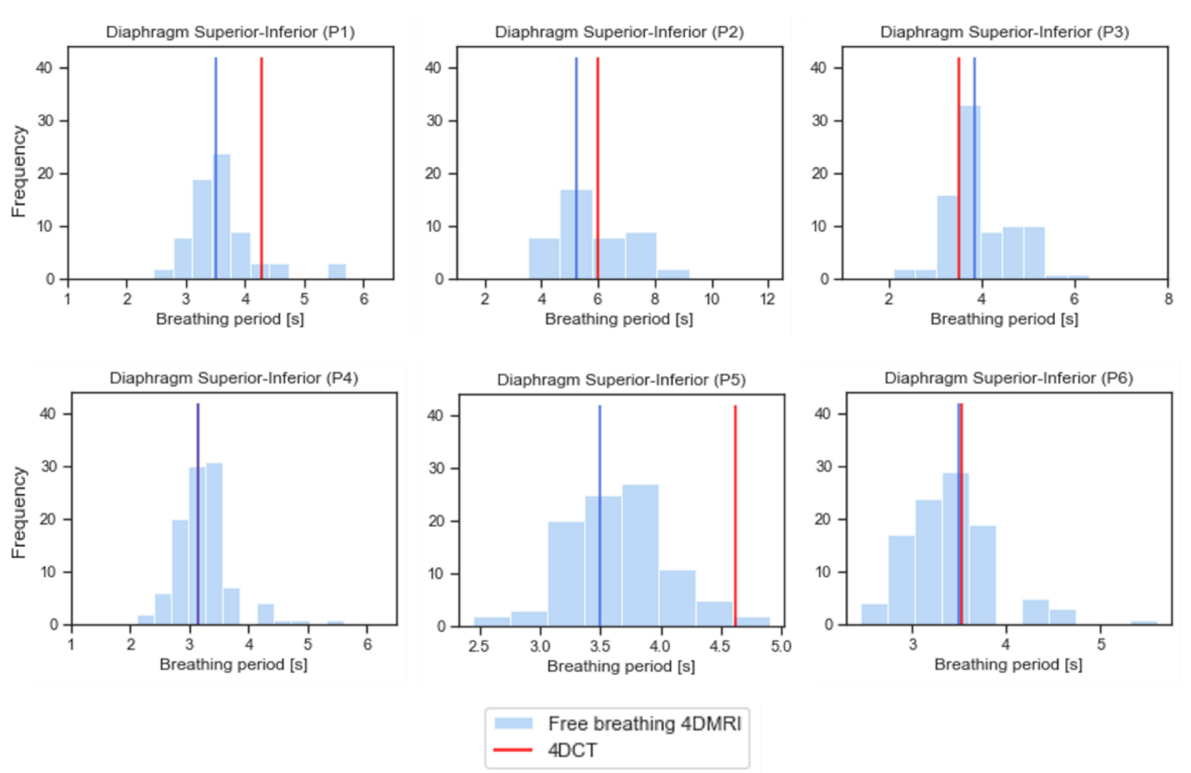


*Figure S.3: breathing period medians from 4DMR acquired over 6-7 minutes and 4DCT.*

|  | **DICE** | **Hausdorff distance [mm]** | **COM**  **distance [mm]** | **∆V%** |
| --- | --- | --- | --- | --- |
| **P1** | 0.90 | 23.27 | 4.19 | 14.53% |
| **P2** | 0.85 | 5.26 | 0.41 | 27.30% |
| **P3** | 0.93 | 4.24 | 1.36 | 8.74% |
| **P4** | 0.82 | 7 | 2.45 | 39.77% |
| **P5** | 0.89 | 9.32 | 1.97 | -4.22% |
| **P6** | 0.93 | 5.37 | 2.07 | 13.9% |

**Table S2:** Similarity indexes between ITV_4DCT_ and ITV_4dMR_.
